# A novel Pd-catalysed sequential carbonylation/cyclization approach toward bis-*N*-heterocycles: rationalization by electronic structure calculations

**DOI:** 10.1098/rsos.181140

**Published:** 2018-09-12

**Authors:** Liliana Damas, Rui M. B. Carrilho, Sandra C. C. Nunes, Alberto A. C. C. Pais, László Kollár, Marta Pineiro, Mariette M. Pereira

**Affiliations:** 1 Coimbra Chemistry Centre, Departamento de Química, Universidade de Coimbra, Rua Larga, 3004-535 Coimbra, Portugal; 2 Department of Inorganic Chemistry, University of Pécs and Szentágothai Research Centre, PO Box 266, 7624 Pécs, Hungary; 3 MTA-PTE Research Group for Selective Chemical Syntheses, Ifjúság u. 6, 7624 Pécs, Hungary

**Keywords:** indole, tandem reactions, carbonylation, cyclization, palladium

## Abstract

An unprecedented palladium-catalysed sequential aminocarbonylation/cyclization synthetic strategy, using carbon monoxide and structurally different aliphatic diamines as *N*-nucleophiles, gives access, in one pot, to a new family of indole-based *N*-heterocyclic derivatives (hydropyrazinones, benzodiazepinones and hydroquinoxalines). Optimization of the reaction conditions towards double carbonylation (*P*_CO_ = 30 bar, *T* = 80°C, iodoindole/diamine ratio = 1 : 1.5, toluene as solvent) allowed the target cyclic products, which are formed *in situ* via intramolecular cyclization of the ketocarboxamide intermediates, to be obtained through a nucleophilic addition/elimination reaction with the pendant terminal amine groups. The structure of the diamine nucleophile was revealed to affect the reaction's selectivity, with the best yields for the cyclic products being obtained in the presence of (1*S*,*2S*)-(+)-cyclohexane-1,2-diamine (**a**) as the nucleophile, using either 5- or 7-iodoindole as the substrate. The reaction's selectivity was rationalized based on electronic structure calculations, which explain the effect of the diamine structure on the predominant formation of the cyclic products.

## 1. Introduction

**Scheme 1.**
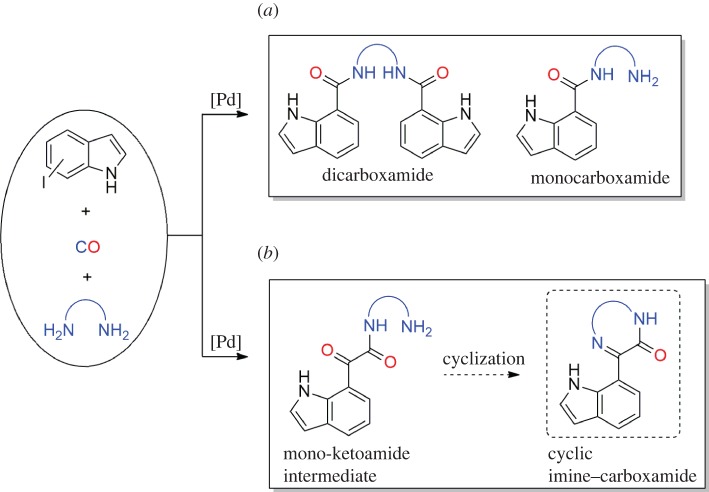
Scope of Pd-catalysed diaminocarbonylation reactions of iodoindoles: (*a*) previous work; (*b*) this work.

Of all the known nitrogen-bearing heterocycles, those containing an indole scaffold hold special relevance in synthetic organic chemistry [1–4]. Scientific interest in this family of alkaloids stems from a number of factors, including their bioactivity and biomedical applications [4–6]. Therefore, the synthesis and functionalization of the indole nucleus has been a topic of increasing interest [7–16]. In some of the recent literature, the synthesis of indole derivatives was reported via direct 2,3-disubstitutions [17,18], C–H activations of the heterocyclic system [19,20] and Pd-catalysed couplings [21–24], while other studies have used oxidations [25] or cyclization [26,27] reactions. In addition, Pd-catalysed carbonylation of halo-indoles [28] along with other *N*-heterocycles, such as halo-quinolines [29] and halo-pyridines [30], using cheap and widely available carbon monoxide as a building block, has been applied as an effective approach to the functionalization of *N*-heterocycles [18,31–33]. More recently, we reported the first Pd-catalysed double carbonylation of 5-iodo- and 7-iodoindole using monoamines as the nucleophiles, which allowed the synthesis of a new family of indole-based ketocarboxamides [34]. We also described the aminocarbonylation of 7-iodoindole in the presence of diamines [34], leading to indole-based mono- and/or dicarboxamides (scheme 1*a*).

In spite of the recent developments in efficient synthetic procedures for complex indole-derived molecules, the synthesis of indole-based pyrazinones and benzodiazepinones, which are commonly found in many natural products and pharmaceuticals, still remains a great challenge [35–38]. Furthermore, their preparation is often accomplished through laborious experimental procedures involving numerous synthetic steps and difficult isolation/purification of intermediates, leading to expensive and low-yielding global synthesis [39–41]. Therefore, prompted by our previous investigation into the potential and synthetic applications of Pd-catalysed aminocarbonylation [34,42–44], the seminal works of Yamamoto's group on the double carbonylation of aryl halides [45–50] and the review on the synthesis of 2-ketoamides in catalytic reactions [51], herein we report a novel one-pot synthetic route towards bis-*N*-heterocyclic derivatives (indole-based hydropyrazinones, benzodiazepinones and hydroquinoxalines), through a sequential Pd-catalysed amino double carbonylation/cyclization strategy, using carbon monoxide and structurally different aliphatic diamines as *N*-nucleophiles. Electronic structure calculations were carried out to rationalize the reaction selectivity for indole-based cyclic molecules with the different amines.

## 2. Experimental

### 2.1. Materials and methods

All moisture-sensitive reagents were manipulated under a nitrogen atmosphere using Schlenk and syringe techniques. Glassware was dried in an oven at 200°C and cooled under a nitrogen atmosphere. Palladium(II) acetate, triphenylphosphine, the diamines and the iodoindole substrates (7-iodoindole and 5-iodoindole) were purchased from Sigma-Aldrich and were used without further purification. Nuclear magnetic resonance (NMR) spectra were recorded on a Bruker Avance 400 spectrometer, operating at 400.13 MHz for ^1^H NMR and 100.62 MHz for ^13^C NMR. Chemical shifts (δ) are reported in ppm relative to CDCl_3_ (7.26 and 77.16 ppm for ^1^H and ^13^C, respectively) or tetramethylsilane (TMS). High-resolution mass spectrometry (HRMS) analysis was carried out on a Bruker Microtof apparatus, equipped with a selective electrospray ionization (ESI) detector. The specific rotation [α] was measured using an electrical polarimeter (Optical Activity AA-5). Melting points, with uncorrected values, were determined with a capillary microscope electrothermal melting point apparatus. The Fourier transform infrared (FT-IR) spectra were measured in KBr pellets using a Thermo Scientific Nicolet 380 spectrometer.

### 2.2. Computational studies

Quantum chemical calculations were carried out in order to rationalize the reaction selectivity with the different amines. The conformations of the different ketocarboxamide intermediates were inspected by performing relaxed potential energy surface scans at the semi-empirical PM3 level, around the dihedrals considered to be more relevant to cyclization. The selected dihedrals were stepped using a step size of 90°. Two different structures of ketocarboxamide intermediate **4b** were explored, one containing the NH_2_ group in the equatorial position and the other with the group substituted in the axial position. Both structures were fully optimized at the density functional theory (DFT) level using the B3LYP functional and the 6-31G(d,p) basis set. Additionally, the structures of the final products, **2a**, **2b, 2c, 2d** and **6a**, were fully optimized at the same level of theory. All the calculations were performed using Gaussian 03 [52] and Gamess [53] program packages. Graphical representations were obtained with Gaussview and Molden 5.0. The potential energy surface scans with the graphical representations obtained for the conformers are presented in the electronic supplementary material.

### 2.3. General procedure of aminocarbonylation reactions

In a typical reaction, Pd(OAc)_2_ (2.8 mg, 0.0125 mmol), triphenylphosphine (6.56 mg, 0.025 mmol), the substrate (0.5 mmol) and the diamine nucleophile (0.75 mmol) were placed in an autoclave. After three cycles of CO/vacuum, Et_3_N (0.25 ml) and toluene (5 ml) were added via a cannula. After three cycles of CO/vacuum, the autoclave was pressurized to the desired CO pressure. The reaction was stirred for 24 h at 80°C. Following this, the autoclave was cooled and slowly depressurized. The mixture was then concentrated and evaporated to dryness with the crude being analysed by NMR spectroscopy. The residue was dissolved in dichloromethane (20 ml) and washed with brine (3 × 20 ml) and water (3 × 20 ml). The organic phase was dried over Na_2_SO_4_, filtered and evaporated to a solid material or to a waxy residue. All compounds were purified by column chromatography (Silicagel 60 (Merck), 0.063–0.200 mm), using EtOAc, EtOAc/*n*-hexane mixtures or ethanol as the eluent (specified below for each compound).

### 2.4. Characterization of products

#### 2.4.1. (4a*S*,8a*S*)-3-(1*H*-indol-7-yl)-4a,5,6,7,8,8a-hexahydroquinoxalin-2(1*H*)-one (**2a**)

R_f_ (70% EtOAc, 30% *n*-hexane) 0.58; brown solid: 57 mg, 43% yield; mp 203–205°C; [α]_D_^20^ = +5.5 (*c* 0.1, CH_2_Cl_2_); ^1^H NMR (CDCl_3_, 400 MHz): *δ* (ppm) = 10.91 (s, 1H), 8.36 (d, *J* = 7.7 Hz, 1H), 7.78 (d, *J* = 7.8 Hz, 1H), 7.29 (t, *J* = 7.6 Hz, 1H), 7.17 (t, *J* = 7.8 Hz, 1H), 6.65 (s, 1H), 6.58 (t, *J* = 7.4 Hz, 1H), 3.39–3.29 (m, 1H), 3.19–3.10 (m, 1H), 2.49 (d, *J* = 7.7 Hz, 1H), 2.00 (d, *J* = 7.7 Hz, 1H), 1.93 (d, *J* = 11.6 Hz, 1H), 1.84 (d, *J* = 12.8 Hz, 1H), 1.59–1.32 (m, 4H); ^13^C NMR (CDCl_3_, 100.6 MHz): *δ* (ppm) = 161.4, 158.6, 134.9, 128.9, 126.1, 124.6, 124.5, 119.2, 117.0, 102.7, 63.1, 53.9, 32.3, 30.9, 25.2, 23.8; IR (KBr, ν (cm^−1^)): 3347, 3186, 1679; HRMS (ESI) *m/z*: calcd for C_16_H_17_N_3_O [M + H]^+^ 268.1444; found: 268.1442.

#### 2.4.2. *N*,*N*-dimethyl-1*H*-indole-7-carboxamide (**2.1**)

R_f_ (75% CHCl_3_, 25% EtOAc) 0.26; dark red solid: 25 mg, 15% yield; mp 100–102°C; ^1^H NMR (TMS, 400 MHz): *δ* (ppm) = 9.42 (sl, 1H), 7.70 (d, *J* = 7.8 Hz, 1H), 7.27–7.21 (m, 2H), 7.08 (t, *J* = 7.6 Hz, 1H), 6.54 (dd, *J* = 3.1, 2.2 Hz, 1H), 3.16 (s, 6H); ^13^C NMR (CDCl_3_, 100.6 MHz): *δ* (ppm) = 170.7, 134.9, 129.2, 125.4, 123.1, 121.5, 118.4, 117.4, 102.4, 39.9–36.1 (br s); elemental analysis: calcd (%) for C_11_H_12_N_2_O (188.2): C 70.2, H 6.43, N 14.88; found C 71.1, H 6.1, N 13.7.

#### 2.4.3. (4a*S*,8a*R*)-3-(1*H*-indol-7-yl)-4a,5,6,7,8,8a-hexahydroquinoxalin-2(1*H*)-one (**2b**)

R_f_ (70% EtOAc, 30% *n*-hexane) 0.58; brown solid: 27 mg, 20% yield; mp 180–184°C; ^1^H NMR (CDCl_3_, 400 MHz): *δ* (ppm) = 10.96 (s, 1H), 8.40 (d, *J* = 7.6 Hz, 1H), 7.78 (d, *J* = 7.8 Hz, 1H), 7.30 (t, *J* = 7.8 Hz, 1H), 7.17 (t, *J* = 7.8 Hz, 1H), 7.15 (br s, 1H), 6.60 (t, *J* = 8.4 Hz, 1H), 4.12–4.05 (m, 1H), 3.71 (br s, 1H), 2.01–1.90 (m, 1H), 1.86 (br s, 1H), 1.78–1.65 (m, 3H), 1.65–1.54 (m, 1H), 1.46 (m, 2H); ^13^C NMR (CDCl_3_, 100.6 MHz): *δ* (ppm) = 160.8, 158.5, 134.9, 128.9, 125.9, 124.5, 124.5, 119.1, 117.3, 102.6, 57.6, 49.0, 29.8, 28.6, 22.6, 21.3; IR (KBr, ν (cm^−1^)): 3350, 3177, 1679; HRMS (ESI) *m/z*: calcd for C_16_H_17_N_3_O [M + H]^+^ 268.1444; found: 268.1442.

#### 2.4.4. 3-(1*H*-indol-7-yl)-5,6-dihydropyrazin-2(1*H*)-one (**2c**)

R_f_ (EtOAc) 0.54; light brown solid: 25 mg, 23% yield; mp 155–157°C; ^1^H NMR (CDCl_3_, 400 MHz): *δ* (ppm) = 10.82 (s, 1H), 8.34 (d, *J* = 7.6 Hz, 1H), 7.78 (d, *J* = 7.9 Hz, 1H), 7.30–7.27 (m, 1H), 7.15 (t, *J* = 7.6 Hz, 1H), 6.69 (br s, 1H), 6.60–6.57 (m, 1H), 4.04 (t, *J* = 6.0 Hz, 2H), 3.54–3.48 (m, 2H); ^13^C NMR (CDCl_3_, 100.6 MHz): *δ* (ppm) = 162.4, 157.8, 134.8, 128.9, 126.0, 124.7, 124.6, 119.1, 117.3, 102.7, 48.4, 39.2; IR (KBr, ν (cm^−1^)): 3323, 1652; HRMS (ESI) *m/z*: calcd for C_12_H_12_N_3_O [M + H]^+^ 214.0975; found 214.0975.

#### 2.4.5. 3-(1*H*-indol-7-yl)-6,7-dihydro-1*H*-1,4-diazepin-2(5*H*)-one (**2d**)

R_f_ (80% EtOAc, 20% *n*-hexane) 0.38; beige solid: 25 mg, 22% yield; mp 173–175°C; ^1^H NMR (CDCl_3_, 400 MHz): *δ* (ppm) = 10.90 (s, 1H), 7.78 (d, *J* = 7.8 Hz, 1H), 7.66 (d, *J* = 7.5 Hz, 1H), 7.31 (t, *J* = 7.4 Hz, 1H), 7.15 (t, *J* = 7.7 Hz, 1H), 6.80 (s, 1H), 6.60 (t, *J* = 7.8 Hz, 1H), 3.93 (br s, 2H), 3.23–3.14 (m, 2H), 2.09–2.03 (m, 2H); ^13^C NMR (CDCl_3_, 100.6 MHz): *δ* (ppm) = 167.8, 167.0, 134.5, 129.0, 125.0, 124.7, 124.7, 119.3, 116.7, 102.5, 48.5, 37.6, 29.1; IR (KBr, ν (cm^−1^)): 3368, 3187, 1667; HRMS (ESI) *m/z*: calcd for C_13_H_13_N_3_O [M + H]^+^ 228.1131; found 228.1132.

#### 2.4.6. *N*,*N′*-(propane-1,3-diyl)bis(2-(1*H*-indol-7-yl)-2-oxoacetamide) (**3d**)

R_f_ (80% EtOAc, 20% *n*-hexane) 0.68; light brown solid: 19 mg, 18% yield; mp 157–159°C; ^1^H NMR (acetone-d6, 400 MHz): *δ* (ppm) = 11.04 (br, 2H), 8.46 (d, *J* = 7.6 Hz, 2H), 8.27 (br, 2H), 7.99 (d, *J* = 7.8 Hz, 2H), 7.49 (d, *J* = 8.8 Hz, 2H), 7.19 (td, *J* = 7.7, 2.6 Hz, 2H), 6.63 (d, *J* = 2.4 Hz, 2H), 3.66–3.52 (m, 4H), 2.01–1.95 (m, 1H), 1.82–1.75 (m, 1H); ^13^C NMR (acetone-d6, 100.6 MHz): *δ* (ppm) = 190.7, 165.3, 136.0, 130.9, 129.4, 129.2, 127.7, 119.6, 117.8, 103.3, 37.2, 32.7; IR (KBr, ν (cm^−1^)): 3392, 3276, 1663, 1633; HRMS (ESI) *m/z*: calcd for C_23_H_20_N_4_NaO_4_+ [M + Na]^+^ 439.1377; found 439.1377.

#### 2.4.7. *N*-(6-aminohexyl)-2-(1*H*-indol-7-yl)-2-oxoacetamide (**4e**)

R_f_ (EtOH) 0.17; brown solid: 89 mg, 62% yield; mp 147–149°C; ^1^H NMR (CDCl_3_, 400 MHz): *δ* (ppm) = 10.41 (br s, 1H), 8.80 (d, *J* = 7.7 Hz, 1H), 7.96 (d, *J* = 7.7 Hz, 1H), 7.32 (t, *J* = 5.8 Hz, 1H), 7.23 (br s, 1H), 7.20 (t, *J* = 7.8 Hz, 1H), 6.65–6.60 (m, 1H), 3.45 (q, *J* = 14.8 Hz, 2H), 1.71–1.64 (m, 4H), 1.51–1.45 (m, 4H), 1.30–1.18 (m, 4H); ^13^C NMR (CDCl_3_, 100.6 MHz): *δ* (ppm) = 188.1, 162.9, 136.0, 132.2, 129.6, 128.8, 125.6, 119.5, 116.9, 103.3, 39.4, 29.3, 26.6; IR (KBr, ν (cm^−1^)): 3429, 1670, 1633; HRMS (ESI) *m/z*: calcd for C_16_H_21_N_3_O_2_ [M + H]^+^ 288.1707; found 288.1706.

#### 2.4.8. (4a*S*,8a*S*)-3-(1*H*-indol-5-yl)-4a,5,6,7,8,8a-hexahydroquinoxalin-2(1*H*)-one (**6a**)

R_f_ (70% EtOAc, 30% *n*-hexane) 0.51; beige solid: 59 mg, 44% yield; mp 147–149°C; [α]_D_^20^ = +2.25 (*c* 0.6, CH_2_Cl_2_); ^1^H NMR (CDCl_3,_ 400 MHz): *δ* (ppm) = 8.34 (s, 1H), 8.29 (s, 1H), 7.78 (dd, *J* = 8.6, 1.5 Hz, 1H), 7.35 (d, *J* = 8.6 Hz, 1H), 7.17 (t, *J* = 7.2 Hz, 1H), 6.60–6.56 (m, 1H), 6.30 (s, 1H), 3.25–3.19 (m, 2H), 2.45 (d, *J* = 11.8 Hz, 1H), 2.01–1.76 (m, 4H), 1.53–1.38 (m, 3H); ^13^C NMR (CDCl_3_, 100.6 MHz): *δ* (ppm) = 162.1, 158.9, 137.2, 127.7, 127.2, 124.9, 122.9, 122.8, 110.8, 104.0, 63.1, 54.2, 32.1, 31.2, 25.3, 23.9; IR (KBr, *ν* (cm^−1^)): 3403, 3269, 1675; HRMS (ESI) *m/z*: calcd for C_16_H_17_N_3_O [M + H]^+^ 268.1444; found: 268.1442.

## 3. Results and discussion

**Scheme 2.**
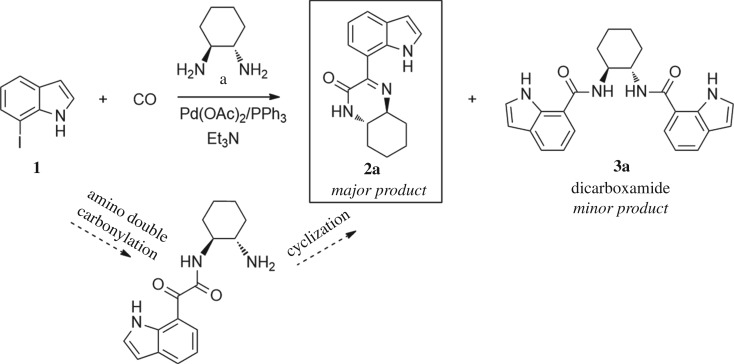
Pd-catalysed amino double carbonylation/cyclization of 7-iodoindole (**1**), using (1*S*,2*S*)-(+)-cyclohexane-1,2-diamine (**a**) as the *N*-nucleophile.

As a starting point, the amino double carbonylation/cyclization reaction of 7-iodoindole was investigated (**1**) with the cycloaliphatic diamine (1*S*,2*S*)-(+)-cyclohexane-1,2-diamine (**a**), in the presence of CO and a Pd(0) catalyst formed *in situ* by the addition of palladium(II) acetate to triphenylphosphine (PPh_3_). Following a standard procedure, the substrate **1** and the diamine nucleophile **a** were introduced in the autoclave, with the palladium precursor, the phosphine ligand and triethylamine (Et_3_N) used as a base (scheme 2).

**Scheme 3.**
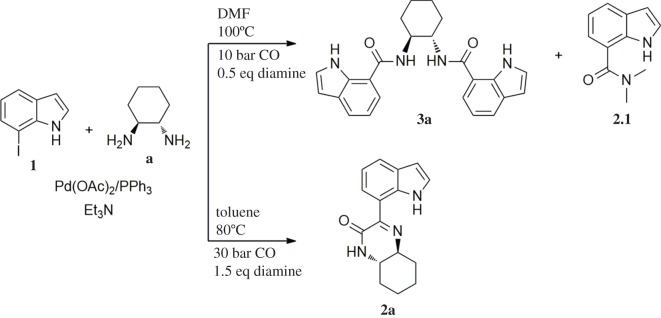
Optimization of the reaction conditions (solvent, temperature, pressure).

The reaction was initially performed in dimethylformamide (DMF), at 100°C and 10 bar CO, using an excess of iodoindole substrate with respect to the diamine (**1**/**a** molar ratio = 2 : 1) (scheme 3). Under these conditions, a full conversion in 24 h was observed by ^1^H NMR analysis of the crude reaction mixture. However, besides a mixture of the expected carboxamide and ketocarboxamide products, the formation of *N*,*N*-dimethyl-1*H*-indole-7-carboxamide (**2.1**) was observed as a side-product (15% isolated yield), which results from DMF *in situ* decomposition [34,54]. This problem was overcome by replacing DMF with toluene in the following experiments. Nevertheless, using toluene under the same reaction conditions (10 bar CO, 100°C, 0.5 eq diamine), the target cyclized product **2a** was barely formed, with the dicarboxamide derivative **3a** being identified as the major product [34]. The reaction was then performed using an excess of diamine with respect to the iodoindole substrate (**1**/**a** molar ratio = 1 : 1.5). However, the dicarboxamide derivative **3a** remained as a major product. An increase in CO pressure from 10 to 30 bar (keeping all other parameters unchanged) resulted in a substantial increase in the target cyclic compound **2a**, evidenced by the relative intensification of the ^1^H NMR signals at *δ* = 8.36 ppm (Ar–H_6_) and *δ* = 10.91 ppm (Ar–NH), and by the ^13^C NMR signals at *δ* = 161.4 ppm and *δ* = 158.6 ppm, typical of C=N and C=O, respectively.

Finally, the decrease in temperature to 80°C resulted in almost exclusive formation of the target product **2a**, showing that the combination of a moderate CO pressure (30 bar), a temperature of 80°C and diamine excess (substrate/diamine molar ratio = 1 : 1.5) is crucial to achieve the predominant formation of **2a**, resulting from the cyclization of the amino-ketocarboxamide intermediate via an intramolecular nucleophilic substitution/elimination reaction between the free NH_2_ group and the keto-functionality. It is worth nothing that the amino-ketocarboxamide intermediate, i.e. the ketocarboxamide with a pendant amino group, could not be detected in the NMR spectra of the crude reaction mixture, which means that the cyclization reaction toward **2a** is highly favoured using diamine **a** as the nucleophile.

In order to demonstrate the synthetic potential of this sequential double amino carbonylation/cyclization strategy, the procedure was applied to other aliphatic diamines and to a differently substituted iodoindole substrate (5-iodoindole (**5**)), under the previously optimized conditions. After work-up, the target products were isolated and purified by column chromatography in silica gel using ethyl acetate/*n*-hexane mixtures as the eluent. The results are presented in table 1.

**Table 1.** Pd-catalysed aminocarbonylation/cyclization of iodoindoles using aliphatic diamines as nucleophiles.^a^

entry	substrate	diamine	major product^b^
1	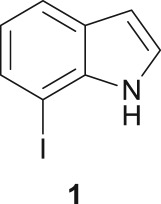	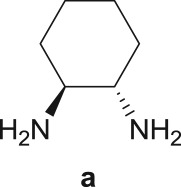	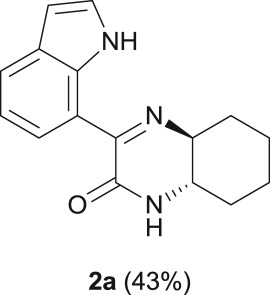
2		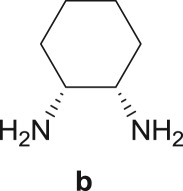	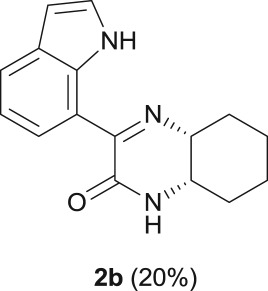
3		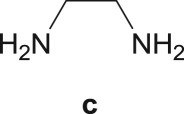	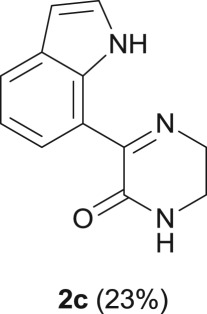
4		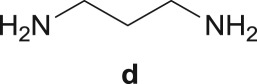	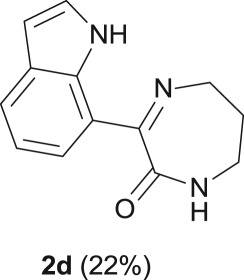
5		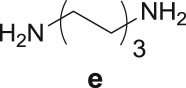	—^c^
6	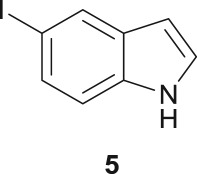	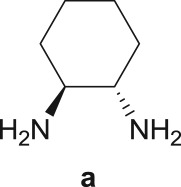	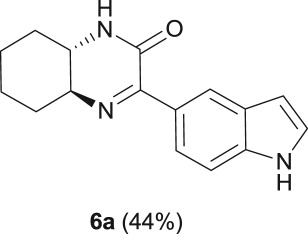

^a^Reaction conditions: substrate (0.5 mmol), diamine (0.75 mmol), Pd(OAc)_2_ (0.0125 mmol), PPh_3_ (0.025 mmol), Et_3_N (0.25 ml), toluene (5 ml); *T* = 80°C, *P*_CO_ = 30 bar, *t* = 24 h.

^b^Isolated yields.

^c^Ketocarboxamide **4e** was obtained (62% isolated yield).

The Pd-catalysed diaminocarbonylation of 7-iodoindole (**1**) was performed using the diastereomeric cycloaliphatic diamines (1*S*,2*S*)-(+)-cyclohexane-1,2-diamine (**a**) and (1*S*,2*R*)-*cis*-cyclohexane-1,2-diamine (**b**), as well as the linear aliphatic diamines such as ethane-1,2-diamine (**c**), propane-1,3-diamine (**d**) and hexane-1,6-diamine (**e**) as the *N*-nucleophiles. The reactions using diamines **a**, **c**, **d** and **e** proceeded with *ca.* 100% conversion, as demonstrated by ^1^H NMR and ^13^C NMR analysis of the crude reaction's mixtures, which showed the shift and disappearance of the proton and carbon resonance signals at *δ* = 8.18 ppm (Ar–NH) ppm and at *δ* = 76.5 ppm (C–I), respectively.

**Figure 1.**
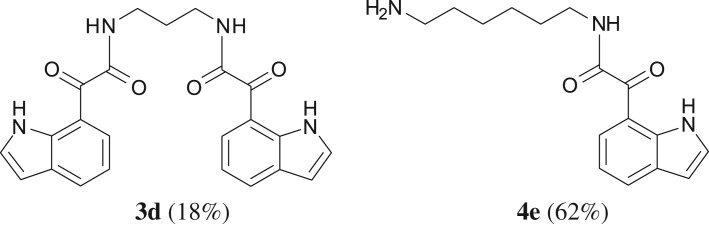
Isolated side products.

When the cyclic diamine **a** was used as the nucleophile, the reaction proceeded with high selectivity for the target cyclic compound **2a**, this being isolated with a 43% yield (table 1, entry 1). However, the reaction with the *cis* diastereoisomer **b** gave significantly lower conversion (56%), with the cyclic product **2b** being isolated with only a 20% yield (table 1, entry 2). The different yields observed with the two diastereoisomers were further analysed on the basis of computational studies (see below). The use of less rigid linear diamines **c** and **d**, containing aliphatic chains with two or three carbon atoms, respectively, led also to *ca.* 100% conversions. Nevertheless, in these cases, a complex mixture of compounds was obtained from which the target cyclization products **2c** (23%) and **2d** (22%) were isolated (table 1, entries 3 and 4). Moreover, the formation of dimeric indole carboxamides and ketocarboxamides as secondary products was confirmed by NMR analysis of the crude reaction mixtures, suggesting that, in these cases, the mono-ketoamide intermediates might undergo a second carbonylation of the free amine, with insertion of an additional iodoindole moiety. However, it was only possible to isolate the bis-ketocarboxamide **3d** with a 18% yield (figure 1). From these results, we conclude that the diamine structure has a significant influence on the reaction selectivity, with the cyclization being favoured with the more rigid cyclic diamine (**a**) and less favoured with linear diamines. In the presence of 1,6-diaminohexane (**e**), the reaction is highly selective towards the double aminocarbonylation, with ketocarboxamide **4e** (figure 1) being predominantly formed and isolated with a 62% yield (table 1, entry 5). However, in this case, the subsequent intramolecular cyclization does not occur, probably because of a less favourable formation of a highly unstable 10-member. Furthermore, analysis of the crude reaction mixture by ^1^H and ^13^C NMR spectroscopy indicated the residual presence of dimeric ketocarboxamides, which were not isolated.

The reaction was further expanded to 5-iodoindole (**5**), using diamine **a** as the nucleophile, and, similar to that observed with 7-iodoindole, the cyclic product **6a**, which was the result of the nucleophilic attack of the amine group to the keto-carbon atom, was obtained with a 44% isolated yield (table 1, entry 6).

### 3.1. Computational studies: rationalization of the reaction's selectivity

**Figure 2.**
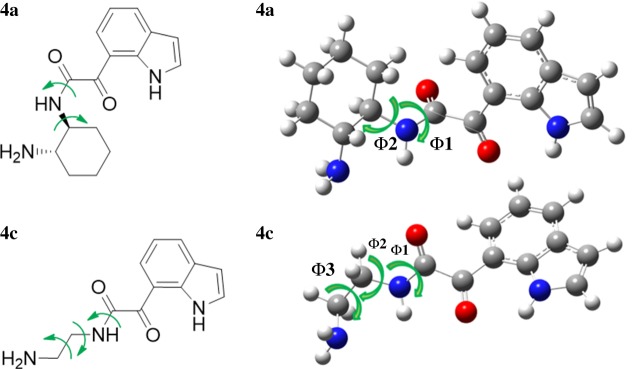
Geometries of ketocarboxamide derivatives **4a** and **4c**, with an indication of the scanned dihedrals. Colour code: grey refers to carbon, red to oxygen, blue to nitrogen and white to hydrogen.

Quantum chemical calculations were carried out to investigate the cyclization step and rationalize the selectivity for the cyclic products with the different diamines. The conformations of the ketocarboxamide intermediates **4a** and **4c** were examined by performing relaxed potential energy surface scans at the semi-empirical PM3 level, around the most relevant dihedrals to the cyclization step (figure 2).

**Figure 3.**
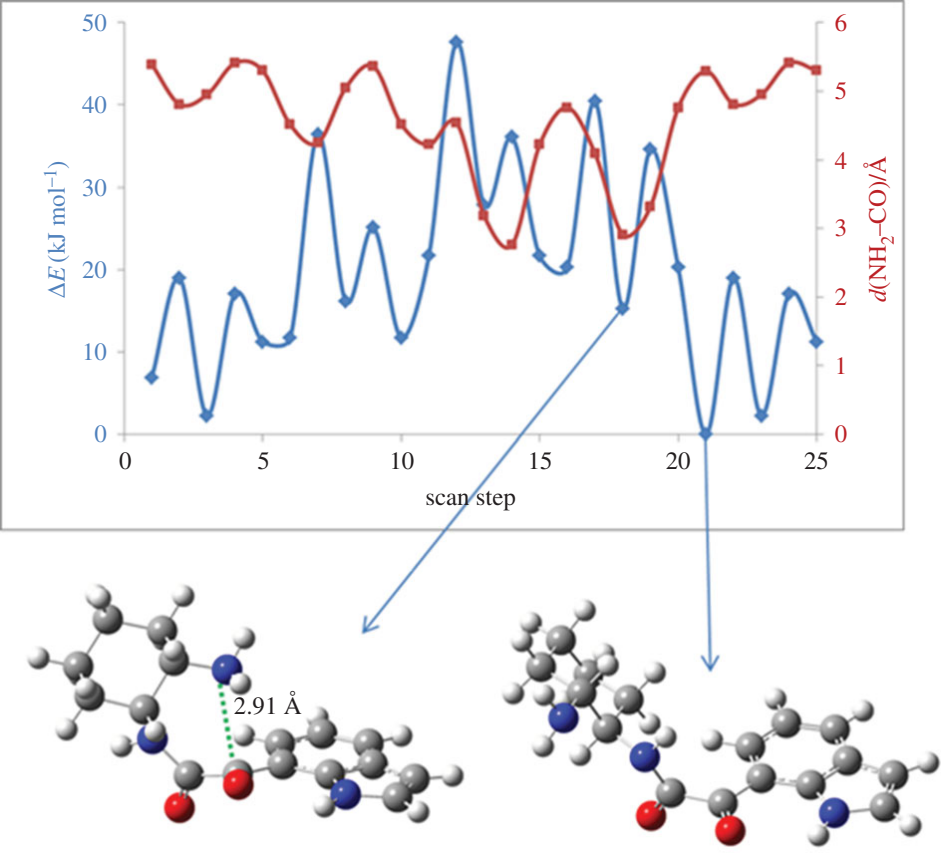
PES of ketocarboxamide intermediate **4a**, indicating the lowest energy conformer (Δ*E* = 0 kJ mol^−1^) and the lowest energy conformer with *d*(NH_2_–CO) < 3 Å, favouring cyclization. PES (blue line) and NH_2_–CO distance (red line) as a function of the scan step (step size = 90°). At each point, all the internal coordinates were relaxed at the PM3 level. Colour code: grey refers to carbon, red to oxygen, blue to nitrogen and white to hydrogen atoms.

The potential energy surface (PES) obtained shows a wide range of conformations with small energy differences. Figure 3 shows the PES obtained for **4a**, while that corresponding to **4c** is included in figure S.1 in the electronic supplementary material. The higher flexibility of diamine **c** leads to an increased number of possible conformations and a higher energy penalty for bringing together the groups involved in the cyclization step (NH_2_ and CO) (figure S.1 in the electronic supplementary material).

An estimation of the energy barrier for the cyclization was obtained considering, in each case, the energy difference between the global minimum and the lowest energy conformations, in which the groups involved in cyclization are closer to each other (*d*(N_NH___2__–C_CO_) ≤ 3 Å).

The results show that the lowest energy conformers are, for the two ketocarboxamide intermediates, those with the more distended backbones in which the groups involved in the cyclization step are away from each other. In comparison with the global minimum, the proximity of the NH_2_ and CO groups requires a change in **Φ1** from 150° to −30° and **Φ2** from −60° to −150° in **4a**; and a change from −30° to 60° in **Φ1** in **4c**. In this case, **Φ2** and **Φ3** remain unaltered at −90° and 60°, respectively. These rotations lower the NH_2_–CO distance from 5.29 to 2.91 Å in **4a** and from 3.78 to 2.99 Å in **4c**. An estimation of the folding penalty can thus be obtained by considering the energy difference between the global minimum and the lowest energy conformations in which the NH_2_ and CO groups are close to each other. Accordingly, an estimation of the energy barrier for cyclization is 15.3 kJ mol^−1^ in **4a** and 49.3 kJ mol^−1^ in **4c**. These findings are in agreement with the results obtained experimentally in which the higher isolated yield was obtained for the cyclic product resulting from the reactions of the iodoindoles with the more rigid cyclic diamine **a** (43% isolated yield of **2a**), while the reaction between 7-iodoindole and diamine **c** afforded the cyclic product **2c** with just 23% isolated yield (table 1, entry 3).

**Scheme 4.**
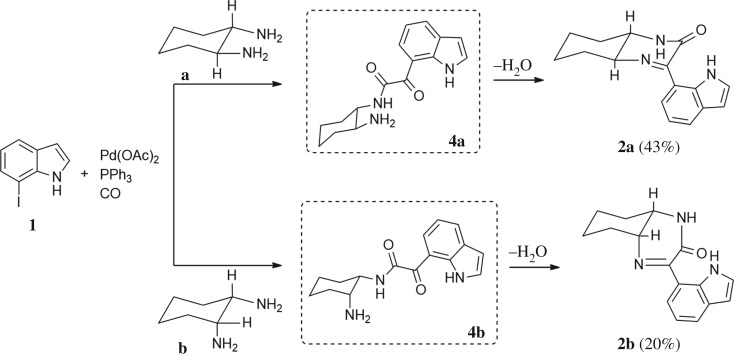
Effect of diamine stereoconfiguration in Pd-catalysed amino double carbonylation/cyclization of 7-iodoindole (**1**), using (1*S*,2*S*)-(+)-cyclohexane-1,2-diamine (**a**) or (1*S*,2*R*)-*cis*-cyclohexane-1,2-diamine (**b**) as nucleophiles.

Another aspect that we explored, using PES scans, was the viability of the cyclization process to occur from **4b** (*cis* diastereoisomer) (scheme 4). To this end, and following the procedure previously described, the results gathered from the PES at the PM3 level for **4a** and **4b** were compared (see figure S.2 in the electronic supplementary material). It was found that for **4a** (*trans* diastereoisomer) the folding of the structure has an energy penalty of *ca* 15 kJ mol^−1^, while the value rises to 41 kJ mol^−1^ for the *cis* diastereoisomer. This result is in good agreement with the experimental results, in which higher isolated yields were obtained with the *trans* diastereoisomer (43% of **2a**) than with the *cis* diastereoisomer (20% of **2b**) (scheme 4).

**Figure 4.**
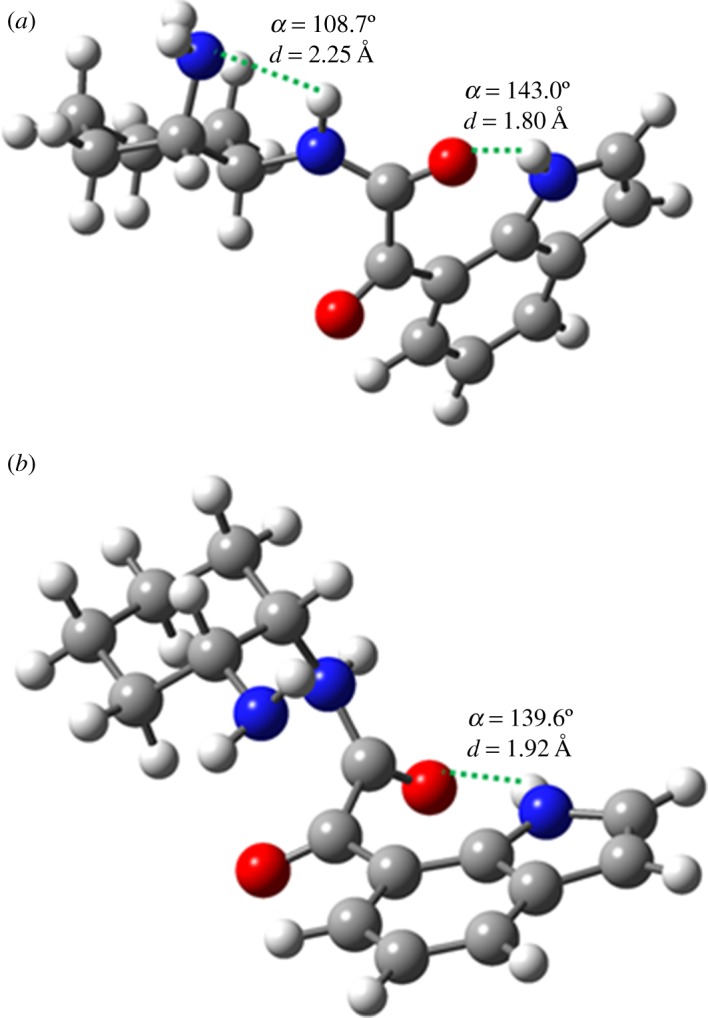
Optimized geometries at the B3LYP/6-31G(d,p) level of the *cis* diastereoisomer (**4b**) with the NH_2_ group in the axial position (*a*) and in the equatorial position (*b*). Colour code: grey refers to carbon, red to oxygen, blue to nitrogen and white to hydrogen atoms.

Still regarding the cyclization of the *cis* diastereoisomer towards the final cyclic product **2b**, further studies were performed in order to evaluate and determine the most favourable position of the NH_2_ group involved in the cyclization process. Thus, two different structures of ketocarboxamide intermediate **4b**, one containing the free NH_2_ group in the equatorial position and the other with this group in the axial position, were optimized at the DFT level, using the B3LYP functional and the 6-31G(d,p) basis set (figure 4). Full optimization showed that the structure with the free NH_2_ group in the axial position (figure 4*a*) is (50 kJ mol^−1^) more stable than that with the free amine group in the equatorial position (figure 4*b*). Both structures show the possibility of hydrogen bond occurrence between the indole NH and the CO groups, with the orientation of both groups and the donor–acceptor distance being slightly more favourable for the *cis* diastereoisomer (**4b**) with the NH_2_ group in the axial position than when it is in the equatorial position. This structure additionally benefits from the possibility of establishing another H-bond between the indole NH and the NH_2_ groups of (1*S*,2*S*)-(+)-cyclohexane-1,2-diamine (**a**). The parameters characterizing each H-bond are depicted in figure 4.

### 3.2. Characterization of the final products

**Figure 5.**
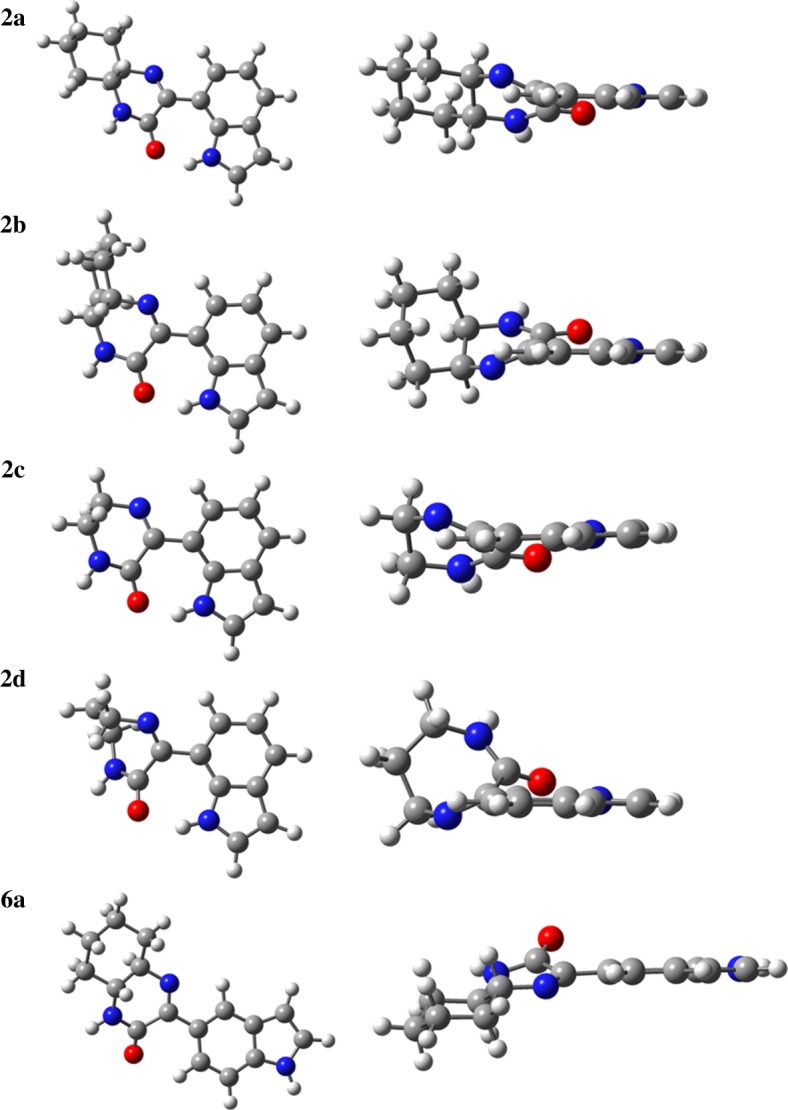
Optimized geometries at the B3LYP/6–31G(d,p) level of the final products **2a**, **2b**, **2c**, **2d** and **6a**. Right column shows the molecules oriented with the indol moiety in plane. Colour code: grey refers to carbon, red to oxygen, blue to nitrogen and white to hydrogen atoms.

The structures of the new indole-based imine–amide cyclic compounds (**2a**, **2b**, **2c**, **2d**, **6a**) (table 1) were fully optimized at the DFT level, using the B3LYP functional and the 6-31G(d,p) basis set. The resulting structures are presented in figure 5.

**Figure 6.**
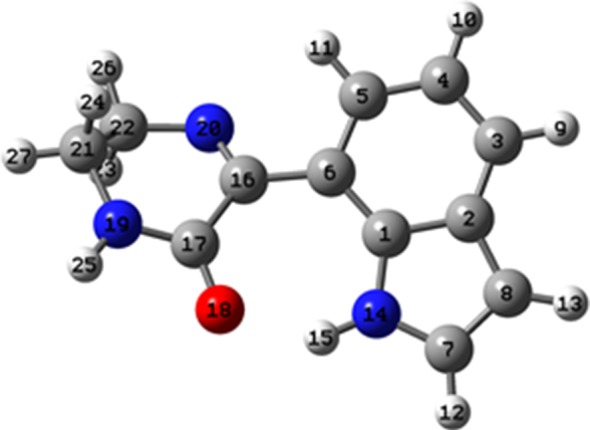
Atom numbering for the common part of the cyclic products optimized at the DFT level.

The structures of the cyclic products are characterized, mostly, by an almost planar arrangement of the imine–amide cyclic moieties relative to the indole group. Moreover, the possibility of a hydrogen bond between the indole NH group and the oxygen atom of the imine-amide moiety is demonstrated. The values of the dihedral angles that define the orientation of the imine–amide cyclic moieties relative to the indole group are compiled in table 2, as well as the bond orders and the parameters characterizing the H-bonds established in each molecule. Figure 6 includes the atom numbering of compound **2c**.

**Table 2.** Structural parameters characterizing the optimized structures of the cyclic products presented in figure 5. Atom numbering is given in figure 6.

	structure				
	**2a**	**2b**	**2c**	**2d**	**6a**
*dihedrals*					
C_5_C_6_C_16_N_20_/°	24.8	−22.2	25.9	−28.3	−16.0
C_6_C_16_N_20_C_22_/°	−173.6	176.2	−173.3	175.3	178.7
C_16_N_20_C_22_C_21_/°	36.6	−36.5	35.2	−75.7	35.7
C_5_C_6_C_16_C_17_/°	−152.9	155.3	−151.9	148.7	161.4
C_6_C_16_C_17_N_19_/°	152.9	−156.3	153.6	−121.5	164.3
*H-bonds*					
*α*(N_14_H_15_O_18_)/°	141.9	142.2	141.4	142.5	—
*d*(H_15_….O_18_)/Å	1.79	1.78	1.80	1.85	—
*bond order*					
C_6_–C_16_	1.018	1.017	1.017	1.019	1.037
C_16_–N_20_	1.691	1.686	1.712	1.740	1.703
C_16_–C_17_	0.898	0.902	0.900	0.870	0.894
C_17_–N_19_	1.141	1.146	1.141	1.157	1.092

The results presented in table 2 show that compound **2d,** with a seven-atom ring, is the one with a more pronounced deviation from planarity. By contrast, **6a** is the most planar, thus favouring some degree of electron delocalization to the C_6_–C_16_ bond and increasing the respective bond order. In this structure, the different position of the imine–amide cyclic moieties in the indole group prevents the occurrence of intramolecular H-bonds, present in all the other structures.

Regarding structures **2a** and **2b**, which result from ketocarboxamide intermediates **4a** and **4b**, respectively, despite the similarity in the parameters reported in table 2, figure 5 shows that the orientation of the cyclohexyl ring relatively to the imine–amide groups is quite different. In fact, structure **2a,** in which the NH groups are both in the equatorial positions, is globally a much more planar structure than **2b**, and *ca.* 4 kJ mol^−1^ more stable than the latter.

## 4. Conclusion

We have implemented a new one-pot Pd-catalysed aminocarbonylation/intramolecular cyclization methodology, which provided an efficient, atom-economic and versatile synthetic strategy to gain access to a family of new cyclic/bicyclic indole-based imine–carboxamide derivatives with unprecedented structures. Our investigation shows that indole-based bis-*N*-heterocycles (hydropyrazinones, benzodiazepinones and hydroquinoxalines) are obtained under optimized conditions towards double carbonylation (*P*_CO_ = 30 bar, *T* = 80°C, iodoindole/diamine ratio = 1 : 1.5, toluene as solvent), via an intramolecular cyclization of the ketocarboxamide intermediates, by the nucleophilic addition/elimination of the terminal amine to the keto-carbon atom, which is favoured using the rigid cyclic diamine (1*S*,2*S*)-(+)-cyclohexane-1,2-diamine (**a**) as the nucleophile. Quantum chemical calculations showed that, using cyclic diamine (**a**) as the nucleophile, the lowest energy conformers, in which the NH_2_ and CO groups are close to each other, have lower energy barriers for cyclization (less than 18 kJ mol^−1^). Instead, if a linear aliphatic diamine is used, the energy barrier for cyclization increased to 49 kJ mol^−1^, which corroborates the low isolated yield obtained experimentally.

The versatility of this novel sequential synthetic approach is demonstrated by the set of aliphatic diamines and the iodo-substituted indole derivatives in different positions. Therefore, it can be regarded as a promising tool for preparation of value-added indole-based molecules, whose bioactivity is currently under evaluation.

## Supplementary Material

Computational data;NMR spectra

## Data accessibility

The datasets supporting this article have been uploaded as part of the electronic supplementary material.
