# Supplementary material for: A novel Pd-catalysed sequential carbonylation/cyclization approach toward bis-N-heterocycles: rationalization by electronic structure calculations
Source: R Soc Open Sci. 2018 Sep 12;5(9):181140. doi: 10.1098/rsos.181140 (PMC6170558; doi:10.1098/rsos.181140)
Supplement: Computational data;NMR spectra [file rsos181140supp1.docx]

**Supporting Information**

**A novel Pd-catalysed sequential carbonylation/cyclisation approach toward bis-*N*-heterocycles: rationalisation by electronic structure calculations**

Liliana Damas,^§^ Rui M. B. Carrilho,^§^ Sandra C. C. Nunes,^§^ Alberto A. C. Pais,^§^ László Kollár,^‡†^ Marta Pineiro,^§*^ and Mariette M. Pereira^§*^

^§^ Coimbra Chemistry Centre, Department of Chemistry, Faculty of Sciences and Technology, University of Coimbra, Rua Larga 3004-535, Coimbra, Portugal

^‡^ Department of Inorganic Chemistry, University of Pécs and Szentágothai Research Centre, PO Box 266, H-7624 Pécs, Hungary

^†^ MTA-PTE Research Group for Selective Chemical Syntheses, Ifjúság u. 6., H-7624 Pécs, Hungary

* Corresponding authors: mpineiro@qui.uc.pt; mmpereira@qui.uc.pt

Table of Contents

1. Computational data..……….…………….………………….……………….………....S1
   1. Potential energy surface scans…….………………………….…….…….………S1
   2. Cartesian coordinates (Å) obtained from the B3LYP/6-31G(d,p) optimizations..……………..…….…….…….…….…….…….……….…………...S4
2. NMR Spectra……………..…….…….…….…….…….…….……….………………..S12
3. References………………………………………………………………………………S20
   - - 1. **Computational data**

Electronic structure calculations were carried out to rationalize the reaction selectivity with the different amines. The conformations of the different ketocarboxamides intermediates were inspected performing relaxed potential energy surface scans at the semi-empirical PM3 level, around the dihedrals considered to be more relevant to cyclization. The selected dihedrals were stepped using a step size of 90°. Two different structures of ketocarboxamide intermediates were explored, one containing the NH_2_ group in equatorial position (**4a**) and the other with the group substituted in the axial position (**4b**). Both structures were fully optimized at the DFT level using the B3LYP functional and the 6-31G(d,p) basis set. Additionally, the structure of the final products **2a**, **2b**, **2c**, **2d** and **6a** were also fully optimized at the same level of theory. All the calculations were performed using Gaussian 03 (1) and Gamess (2) program packages. Graphical representations were obtained with Gaussview and Molden 5.0.

**1.1. Potential energy surface scans**

The conformations of the different ketocarboxamides intermediates were inspected performing relaxed potential energy surface scans at the semi-empirical PM3 level, around the dihedrals considered to be more relevant to cyclization. The selected dihedrals were stepped using a step size of 90°. All the calculations were performed using Gaussian 03^1^ and Gamess^2^ program packages. Graphical representations were obtained with Gaussview and Molden 5.0. The potential energy surface scans with indication of the structures analyzed are presented below.

**Figure S.1.** PES of the ketocarboxamide intermediate **4c** with the indication of lowest energy conformer (∆E=0 kJ/mol) and the lowest energy conformer with the distance between NH_2_-CO favoring cyclization. PES (blue curve) and NH_2_-CO distance (red curve) as a function of scan step (step size = 90°). At each point all the internal coordinates were relaxed at the PM3 level.Color code: gray refers to carbon, red to oxygen, blue to nitrogen and white to hydrogen atoms.

**Figure S.2.** PES of ketocarboxamide intermediate **4a** (top panel) and **4b** (bottom panel) with the indication of lowest energy conformer that minimizes the NH_2_-CO distance, favouring cyclization. PES (blue curve) and NH_2_-CO distance (red curve) as a function of scan step (step size = 90°). At each point all the internal coordinates were relaxed at the PM3 level. Color code: gray refers to carbon, red to oxygen, blue to nitrogen and white to hydrogen atoms.

**1.2 Cartesian coordinates (Å) obtained from the B3LYP/6-31G(d,p) optimizations for final products 2a, 2b, 2c, 2d and 6a (Figure S.3).**

The structures of the final products were fully optimized at the DFT level using the B3LYP functional and the 6-31G(d,p) basis set. Additionally, the structure of the final products **2a**, **2b, 2c, 2d** and **6a** were also fully optimized at the same level of theory (Figure S.3).

**Figure S.3.** Structure of the final of the final products (**2a**, **2b**, **2c**, **2d**, **6a**) which were fully optimized at the DFT level, using the B3LYP functional and the 6-31G(d,p) basis set.

Table 1. Cartesian coordinates (Å) obtained from the B3LYP/6-31G(d,p) optimizations for final products **2a** (Figure S.3).

| C | 2.6110735428 | -0.2596092296 | -0.0863175098 |
| --- | --- | --- | --- |
| C | 3.8941591146 | 0.3433496907 | 0.1274764991 |
| C | 3.9765692629 | 1.6643232527 | 0.5833993573 |
| C | 2.7994212257 | 2.3682904835 | 0.8154174141 |
| C | 1.5518167304 | 1.7758376525 | 0.5888199552 |
| C | 1.4046651745 | 0.4541751393 | 0.1302322697 |
| C | 4.1996830814 | -1.7631322347 | -0.6056069185 |
| C | 4.8808139435 | -0.6416994518 | -0.2127260479 |
| H | 4.9446427749 | 2.1270564924 | 0.7570585908 |
| H | 2.8414335699 | 3.3904851760 | 1.1799986211 |
| H | 0.6464919269 | 2.3448610233 | 0.7647231291 |
| H | 4.5728920579 | -2.7238531402 | -0.9317319923 |
| H | 5.9543000133 | -0.5228962753 | -0.1736085276 |
| N | 2.8427124927 | -1.5319027650 | -0.5431504157 |
| H | 2.0808318247 | -2.1972877716 | -0.6490632084 |
| C | 0.0072245371 | -0.0100772681 | -0.1041963200 |
| C | -0.3902620019 | -1.4879840212 | -0.0038550229 |
| O | 0.3558675068 | -2.4451550120 | -0.2307363224 |
| N | -1.6891329369 | -1.6882557768 | 0.3312917430 |
| N | -0.8865215693 | 0.8845103642 | -0.3554476517 |
| C | -2.6368732608 | -0.6021333081 | 0.5538002342 |
| C | -4.0914816600 | -1.0578930428 | 0.4094093025 |
| C | -2.2898362671 | 0.4962839760 | -0.4532217696 |
| C | -5.0374049263 | 0.1423993171 | 0.5887560406 |
| H | -4.3177493807 | -1.8411025759 | 1.1450769916 |
| C | -3.2116533604 | 1.7067035711 | -0.2644857013 |
| C | -4.6881323795 | 1.2909507319 | -0.3747134477 |
| H | -4.9682223576 | 0.5022198452 | 1.6246075804 |
| H | -3.0144658654 | 2.1500219671 | 0.7208679994 |
| H | -4.8978527666 | 0.9715763600 | -1.4054568139 |
| H | -2.4590262123 | 0.0859143899 | -1.4639141070 |
| H | -2.4985713875 | -0.1918482100 | 1.5684637124 |
| H | -2.9580625727 | 2.4698890249 | -1.0072738932 |
| H | -5.3365678806 | 2.1526255826 | -0.1800288817 |
| H | -6.0744259242 | -0.1782638083 | 0.4395185242 |
| H | -4.2313757748 | -1.5004936213 | -0.5863685848 |
| H | -1.9639802954 | -2.6548865277 | 0.4537851714 |

Table 2. Cartesian coordinates (Å) obtained from the B3LYP/6-31G(d,p) optimizations for final products **2b** (Figure S.3).

| C | 2.6491809442 | -1.3535575661 | 0.7761332446 |
| --- | --- | --- | --- |
| C | 3.4900240279 | -2.4786430284 | 1.0676022030 |
| C | 2.9469673241 | -3.7685570319 | 1.0913519515 |
| C | 1.5906595903 | -3.9284203203 | 0.8279591548 |
| C | 0.7816436995 | -2.8260874599 | 0.5317278740 |
| C | 1.2683385987 | -1.5068207957 | 0.4863631643 |
| C | 4.7437282886 | -0.6095725293 | 1.1180471505 |
| C | 4.8142658227 | -1.9677815595 | 1.2794044194 |
| H | 3.5757845812 | -4.6262071700 | 1.3154919358 |
| H | 1.1453245363 | -4.9187966000 | 0.8522327424 |
| H | -0.2693724836 | -2.9776946304 | 0.3164440582 |
| H | 5.5173358134 | 0.1411915580 | 1.1999109774 |
| H | 5.6989151204 | -2.5418937590 | 1.5156164328 |
| N | 3.4531251514 | -0.2430062917 | 0.8044649033 |
| H | 3.0554451281 | 0.6911676881 | 0.7387225908 |
| C | 0.2841176741 | -0.4557969226 | 0.0901650783 |
| C | 0.4392495121 | 1.0111238809 | 0.5009268790 |
| O | 1.5052934011 | 1.5676597182 | 0.7825734844 |
| C | -1.7966067163 | 0.1243208641 | -0.9343708567 |
| C | -3.0919171508 | -0.6243714834 | -1.2931658965 |
| C | -2.0307350090 | 1.1559471804 | 0.1817307938 |
| C | -3.7846890716 | -1.2184076633 | -0.0590603468 |
| H | -2.8455242983 | -1.4085999607 | -2.0158396158 |
| C | -2.7573889473 | 0.5674754658 | 1.4067179620 |
| C | -4.0597340355 | -0.1370555901 | 0.9962379344 |
| H | -3.1410776588 | -1.9946548906 | 0.3751556237 |
| H | -2.0936019295 | -0.1513653331 | 1.9023451430 |
| H | -4.7676137958 | 0.6002342191 | 0.5895846379 |
| H | -2.9515439055 | 1.3689559949 | 2.1298421498 |
| H | -4.5393275497 | -0.5725221954 | 1.8802823895 |
| H | -4.7182128810 | -1.7107590244 | -0.3551046197 |
| H | -3.7733175099 | 0.0780082837 | -1.7931582714 |
| N | -0.7214553520 | 1.7125503821 | 0.5166520612 |
| H | -2.6378066420 | 1.9736969601 | -0.2275668109 |
| H | -0.6345086114 | 2.6627567121 | 0.8540209161 |
| H | -1.4515187298 | 0.6683412773 | -1.8278947120 |
| N | -0.7490469364 | -0.8322583790 | -0.5816467259 |

Table 3. Cartesian coordinates (Å) obtained from the B3LYP/6-31G(d,p) optimizations for final products **2c** (Figure S.3).

| C | 1.3997569452 | -0.3320033562 | -0.0902005181 |
| --- | --- | --- | --- |
| C | 2.7636463788 | 0.0948095897 | 0.0227046703 |
| C | 3.0535927476 | 1.4369299759 | 0.2970022598 |
| C | 2.0007645486 | 2.3324685616 | 0.4514626472 |
| C | 0.6727927964 | 1.9087590668 | 0.3236442392 |
| C | 0.3189427383 | 0.5763864764 | 0.0458494202 |
| C | 2.7326805822 | -2.1110867940 | -0.4263649336 |
| C | 3.5834099134 | -1.0625909507 | -0.1971295418 |
| H | 4.0842775032 | 1.7685009390 | 0.3925455117 |
| H | 2.2030835876 | 3.3756093277 | 0.6767988442 |
| H | -0.1318034780 | 2.6258986707 | 0.4380195795 |
| H | 2.9496438652 | -3.1509891887 | -0.6263593207 |
| H | 4.6634336897 | -1.1019331732 | -0.1897083465 |
| N | 1.4268900635 | -1.6729081152 | -0.3772633378 |
| H | 0.5742578516 | -2.2267299524 | -0.3717039754 |
| C | -1.1373877486 | 0.3010094115 | -0.1109512822 |
| C | -1.7460663797 | -1.0673310971 | 0.2097413945 |
| O | -1.1535128006 | -2.1471253261 | 0.1330674185 |
| N | -3.0558654736 | -1.0249275938 | 0.5621236456 |
| N | -1.8908606139 | 1.2763268784 | -0.4902619966 |
| C | -3.8296193162 | 0.2081260480 | 0.6162037715 |
| C | -3.3365356471 | 1.0984417027 | -0.5176107855 |
| H | -3.6262936255 | 0.6648734550 | -1.4865107824 |
| H | -3.6956564293 | 0.7146435624 | 1.5831412372 |
| H | -3.4639020369 | -1.9151633905 | 0.8147781123 |
| H | -3.8055685141 | 2.0860422190 | -0.4564365173 |
| H | -4.8917011479 | -0.0304369465 | 0.5060185863 |

Table 4. Cartesian coordinates (Å) obtained from the B3LYP/6-31G(d,p) optimizations for final products **2d** (Figure S.3).

| C | -2.0933378534 | -6.4270680689 | -0.5059936027 |
| --- | --- | --- | --- |
| C | -0.9291942976 | -5.9485783408 | 0.1749959678 |
| C | -0.9827980627 | -5.7175637646 | 1.5565555667 |
| C | -2.1691530936 | -5.9720927637 | 2.2369435364 |
| C | -3.3003481265 | -6.4491667424 | 1.5611898633 |
| C | -3.3059670631 | -6.6851141815 | 0.1775184991 |
| C | -0.4303831207 | -6.2298213139 | -2.0038957421 |
| C | 0.1084756577 | -5.8292259042 | -0.8094075835 |
| H | -0.1084647431 | -5.3479564829 | 2.0858438037 |
| H | -2.2278397707 | -5.7968031505 | 3.3072391503 |
| H | -4.2159022416 | -6.6468560398 | 2.1074809441 |
| H | 0.0220304176 | -6.2896263914 | -2.9839603580 |
| H | 1.1237056166 | -5.4975517218 | -0.6440077313 |
| N | -1.7483964607 | -6.5924104998 | -1.8245020392 |
| H | -2.4120897267 | -6.9157866658 | -2.5237057924 |
| C | -4.5651341090 | -7.1813187732 | -0.4338828047 |
| C | -4.9158620720 | -6.8026639347 | -1.8794130392 |
| C | -6.6645912014 | -8.2800881095 | -0.2902490259 |
| C | -7.6726646326 | -7.1136359228 | -0.3353413883 |
| H | -7.0693881976 | -9.0881595932 | 0.3263219390 |
| H | -6.5457012417 | -8.6831644096 | -1.3063113873 |
| C | -7.0222684762 | -5.8488114057 | -0.9260233986 |
| H | -8.5386806631 | -7.4256633565 | -0.9310651658 |
| H | -8.0354830853 | -6.8779560080 | 0.6713602695 |
| H | -6.4727100212 | -5.3179794890 | -0.1425013759 |
| H | -7.7755159207 | -5.1564451717 | -1.3094243138 |
| N | -5.3781661481 | -7.8788751058 | 0.2776232109 |
| N | -6.0970903124 | -6.1474456965 | -2.0199172350 |
| O | -4.2202832310 | -7.0964089843 | -2.8559403240 |
| H | -6.3648978192 | -5.9587620074 | -2.9775304431 |

Table 5. Cartesian coordinates (Å) obtained from the B3LYP/6-31G(d,p) optimizations for final products **6a** (Figure S.3).

| C | 2.3923819062 | -0.1230735775 | 0.4529948107 |
| --- | --- | --- | --- |
| C | 3.6708945920 | 0.3258352255 | 0.7498317837 |
| C | 3.8799764236 | 1.7066262514 | 0.8282719996 |
| C | 2.8246574926 | 2.6406221645 | 0.6207845028 |
| C | 1.5429884613 | 2.1608039602 | 0.3333623538 |
| C | 1.3172537205 | 0.7815407619 | 0.2426372907 |
| H | 0.7103157473 | 2.8379250666 | 0.1773785469 |
| C | -0.0695313518 | 0.3188313438 | -0.0277707900 |
| C | -0.2999707316 | -1.1038205461 | -0.5546075527 |
| O | 0.5763013960 | -1.7904965190 | -1.0703487324 |
| N | -1.5903971406 | -1.5344221491 | -0.4471824667 |
| N | -1.0445219056 | 1.1427460755 | 0.1386092375 |
| C | -2.6334604334 | -0.7615431703 | 0.2153000016 |
| C | -4.0407980812 | -1.2176697109 | -0.1813589039 |
| C | -2.4067657399 | 0.7069544187 | -0.1519529561 |
| C | -5.0979870127 | -0.3317733852 | 0.5000592176 |
| H | -4.1886728456 | -2.2711401546 | 0.0915858741 |
| C | -3.4328948662 | 1.5980327980 | 0.5578498296 |
| C | -4.8652628421 | 1.1623922289 | 0.2099528563 |
| H | -5.0618503983 | -0.5006407375 | 1.5853958394 |
| H | -3.2688254694 | 1.5321639044 | 1.6420502047 |
| H | -5.0524583922 | 1.3553695715 | -0.8561428372 |
| H | -2.5620713030 | 0.8040579790 | -1.2409642658 |
| H | -2.5324028247 | -0.8569067037 | 1.3108077774 |
| H | -3.2553483799 | 2.6416178505 | 0.2773890145 |
| H | -5.5900778193 | 1.7685227942 | 0.7656323093 |
| H | -6.0998370985 | -0.6318158174 | 0.1724524292 |
| H | -4.1394569724 | -1.1518878976 | -1.2733444954 |
| H | -1.7495572234 | -2.4975541499 | -0.7147366227 |
| C | 3.3951394976 | 3.9543868521 | 0.7705082056 |
| N | 5.0212017446 | 2.4315166916 | 1.0893204605 |
| C | 4.7228727052 | 3.7855495293 | 1.0524279953 |
| H | 2.8796701375 | 4.8999198885 | 0.6803637311 |
| H | 5.4952679992 | 4.5194582903 | 1.2338375195 |
| H | 5.9319542535 | 2.0412053729 | 1.2679828347 |
| H | 2.2116463077 | -1.1854336897 | 0.3661041384 |
| H | 4.4811264471 | -0.3799008106 | 0.9109188583 |

Table 6. Cartesian coordinates (Å) obtained from the B3LYP/6-31G(d,p) optimizations for ketocarboxamide intermediate **4b** with the free NH_2_ group in equatorial position.

| C | 2.8385802644 | -1.2802602950 | 0.5651557227 |
| --- | --- | --- | --- |
| C | 3.8209441691 | -2.1674494797 | 1.1069400947 |
| C | 3.7576974809 | -3.5348759239 | 0.8059230863 |
| C | 2.7513401731 | -3.9977317793 | -0.0374408762 |
| C | 1.7940455129 | -3.1191069008 | -0.5605328738 |
| C | 1.7898059312 | -1.7499207492 | -0.2594831851 |
| C | 4.3164570693 | -0.0657177354 | 1.7481385027 |
| C | 4.7380443012 | -1.3628611718 | 1.8650296739 |
| H | 4.4956401842 | -4.2204106261 | 1.2143285540 |
| H | 2.7008433382 | -5.0527049116 | -0.2908873726 |
| H | 1.0080008240 | -3.4880759460 | -1.2110366588 |
| H | 4.7422520150 | 0.8421297072 | 2.1520002733 |
| H | 5.6032259841 | -1.7085164291 | 2.4124855084 |
| N | 3.1681366354 | -0.0121614231 | 0.9824661029 |
| H | 2.7362044227 | 0.8169627538 | 0.5893966391 |
| C | 0.6545926364 | -0.9539156402 | -0.7969190030 |
| C | 0.4154606620 | 0.4815992598 | -0.3004117782 |
| O | 1.3010883410 | 1.2991115676 | -0.5929406544 |
| N | -0.7551257263 | 0.9318651156 | 0.2311781606 |
| C | -2.2584591813 | -1.0467651977 | 0.8615223951 |
| C | -2.9597650103 | -1.1709064624 | -0.4990513420 |
| C | -1.7968134355 | 0.3948374909 | 1.1369093851 |
| C | -4.1520941392 | -0.2046668711 | -0.5906307248 |
| H | -3.3056747684 | -2.2053017664 | -0.6270211267 |
| C | -3.0080156776 | 1.3457878044 | 1.0599630820 |
| C | -3.7350850576 | 1.2434768862 | -0.2899873446 |
| H | -4.9298232014 | -0.5118148145 | 0.1239795384 |
| H | -3.6954701042 | 1.0861443140 | 1.8754466267 |
| H | -3.0720543946 | 1.6039126614 | -1.0861963159 |
| H | -3.0238799191 | -1.2387689787 | 1.6371854989 |
| H | -2.6847726200 | 2.3775011303 | 1.2521451988 |
| H | -4.6122002426 | 1.9008922857 | -0.2864856135 |
| H | -4.6042988488 | -0.2668595115 | -1.5869862652 |
| H | -2.2382506497 | -0.9749867466 | -1.2974680738 |
| H | -0.6809411499 | 1.9431545553 | 0.3069369723 |
| H | -1.3925349859 | 0.4225349742 | 2.1618015544 |
| N | -1.1181265835 | -1.9545239569 | 0.9867143779 |
| H | -0.8984174145 | -2.1384350234 | 1.9620077708 |
| H | -1.3067006570 | -2.8486448934 | 0.5423567734 |
| O | -0.0081561778 | -1.3183272724 | -1.7598322839 |

Table 7. Cartesian coordinates (Å) obtained from the B3LYP/6-31G(d,p) optimizations for ketocarboxamide intermediate **4b** with the free NH_2_ group in axial position.

| C | 1.9263631001 | -3.9975494159 | 2.3413808407 |
| --- | --- | --- | --- |
| C | 2.8259554133 | -5.0276349926 | 1.9123265894 |
| C | 2.3252242109 | -6.1449156299 | 1.2344149104 |
| C | 0.9542282583 | -6.2429293984 | 1.0019878139 |
| C | 0.0838074044 | -5.2359883296 | 1.4274954871 |
| C | 0.5318418339 | -4.0787366466 | 2.0920739192 |
| C | 4.0000962643 | -3.4489331512 | 3.0056908607 |
| C | 4.1387253739 | -4.6405091758 | 2.3450712768 |
| H | 2.9985100584 | -6.9298616116 | 0.8994605047 |
| H | 0.5539220604 | -7.1073589532 | 0.4809330717 |
| H | -0.9807757406 | -5.3174971287 | 1.2375291348 |
| H | 4.7440190482 | -2.8318635560 | 3.4895570887 |
| H | 5.0590950021 | -5.1857204835 | 2.1914728718 |
| N | 2.6756529141 | -3.0618788485 | 3.0004616156 |
| H | 2.2433183742 | -2.2641338579 | 3.4643885633 |
| C | -0.5136627732 | -3.0731572811 | 2.4000609988 |
| C | -0.1475297259 | -1.6186869754 | 2.7654904675 |
| O | 0.6827517620 | -1.3804988456 | 3.6551376453 |
| N | -1.9251687095 | 1.7712890202 | 1.3925665775 |
| C | -1.8084712993 | -0.6713607194 | 1.0703255811 |
| C | -1.1489048427 | -0.6944290793 | -0.3235641692 |
| C | -2.7417434743 | 0.5523470264 | 1.2438662282 |
| C | -2.2051175377 | -0.6575832299 | -1.4402172546 |
| H | -0.5247028557 | -1.5912448835 | -0.4108694806 |
| C | -3.7821592682 | 0.5873225618 | 0.1045354717 |
| C | -3.1340109524 | 0.5560641665 | -1.2886065529 |
| H | -2.8036576471 | -1.5785151629 | -1.4014108100 |
| H | -4.4424268075 | -0.2844848953 | 0.2110098704 |
| H | -2.5582250250 | 1.4788109384 | -1.4553166657 |
| H | -2.4113297260 | -1.5728461556 | 1.1937153958 |
| H | -4.4171180124 | 1.4749411603 | 0.2193174144 |
| H | -3.9123114583 | 0.5459244097 | -2.0603024741 |
| H | -1.7148803059 | -0.6460439685 | -2.4204537445 |
| H | -0.4698099056 | 0.1641198955 | -0.4200207374 |
| H | -2.4860519504 | 2.5204868620 | 1.7886968226 |
| H | -3.2660195368 | 0.4238721791 | 2.1980906803 |
| N | -0.8188821473 | -0.6292631153 | 2.1466055885 |
| H | -0.5647245691 | 0.3149288194 | 2.4320816748 |
| O | -1.7090144213 | -3.3563982673 | 2.3739698301 |
| H | -1.5952123861 | 2.1027167192 | 0.4892470928 |

- - - 1. **NMR Spectra**

**3. References**

(1) Frisch M. J., Trucks G. W., Schlegel H. B., Scuseria G., Robb M. A., Cheeseman J. R., Montgomery Jr J. A., Vreven T., Kudin K. N., Burant J. C., Millam J. M., Iyengar S. S., Tomasi J., Barone V., Mennucci B., Cossi M., Scalmani G., Rega N., Petersson G. A., Nakatsuji H., Hada M., Ehara M., Toyota K., Fukuda R., Hasegawa J., Ishida M., Nakajima T., Honda Y., Kitao O., Nakai H., Klene M., Li X., Knox J. E., Hratchian H. P., Cross J. B., Bakken V., Adamo C., Jaramillo J., Gomperts R., Stratmann R. E., Yazyev O., Austin A. J., Cammi R., Pomelli C., Ochterski J. W., Ayala P. Y., Morokuma K., Voth G. A., Salvador P., Dannenberg J. J., Zakrzewski V. G., Dapprich S., Daniels A. D., Strain M. C., Farkas O., Malick D. K., Rabuck A. D., Raghavachari K., Foresman J. B., Ortiz J. V., Cui Q., Baboul A. G., Clifford S., Cioslowski J., Stefanov B. B., Liu G., Liashenko A., Piskorz P., Komaromi I., Martin R. L., Fox D. J., Keith T., Al-Laham M. A., Peng C. Y., Nanayakkara A., Challacombe M., Gill P. M. W., Johnson B., Chen W., Wong M. W., Gonzalez C., Pople J. A., 2004 GAUSSIAN 03 (Revision D.01), Gaussian Inc., Wallingford, CT,.

(2) Schmidt M. W., Baldridge K. K., Boatz J. A., Elbert S. T., Gordon M. S., Jensen J. H., Koseki S., Matsunaga N., Nguyen K. A., Su S. J., Windus T. L., Dupuis M., Montgomery J. A. 1993 General atomic and molecular electronic structure system. *J. Comput. Chem.* **14**, 134713-63. doi:10.1002/jcc.540141112)
